# Supplementary material for: Systematic review and meta-analysis on the effect of depression on ART adherence among women living with HIV
Source: PLoS One. 2024 Jun 20;19(6):e0300106. doi: 10.1371/journal.pone.0300106 (PMC11189173; doi:10.1371/journal.pone.0300106)
Supplement: Attachment — Submitted filename: Review comment for Plosone.docx [file pone.0300106.s002.docx]

**Reviewer’s Comment**

**Overview**

The manuscript, entitled "Systematic Review and Meta-Analysis on the Impact of Depression on ART Adherence Among Women Living with HIV," is well-written. It addresses a crucial aspect of the HIV program, particularly in the context of improving adherence among women on ART. This work is significant due to its potential to influence strategies and interventions aimed at improving health outcomes for this vulnerable population. I have the following suggestions to improve the manuscript:

**Abstract**

- On page 2, line 34, the authors’ stated objective, “to pool the effect of depression on ART adherence among women living with HIV,” is generally clear but could be improved for precision and clarity. An improved objective might be: “To conduct a meta-analysis on the correlation between depression and adherence to antiretroviral therapy among women living with HIV in Sub-Saharan Africa.”

**Introduction**

- This section effectively introduces the topic and establishes the relevance of studying the effect of depression on ART adherence in women living with HIV. It might benefit from a more concise summary of existing literature and a clearer articulation of the knowledge gap this study addresses.

**Objective**

- On page 7, line 2, the objective is clearly stated and aligns well with the introduction. However, the gap the study was trying to address was not clearly described.
- Authors should ensure that the objective is clear and directly linked to the gaps or issues identified in the introduction. This creates a cohesive narrative from the problem to the study's aim.
- It was unclear why the authors stated the objective separately rather than integrating it with the introduction section. Authors should review journal guidelines and ensure this format aligns with journal requirements.

**Method**

- This section describes the systematic review process, including database search strategies and criteria for inclusion and exclusion of studies. It is detailed and provides a clear understanding of the methodology. However, in the introduction, (page 4, lines 86), the authors described the challenges of defining the etiology of the exposure variable. Therefore, it is important to be sure that all articles are describing the same exposure variable.
- The exposure variable “depression” was not well defined for the studies selected. A table summarizing the definition of depression for each of the eight articles studied will ensure that we are not comparing “apples with oranges.”

**Data Analysis**

The manuscript uses a fixed-effect model for the meta-analysis and employs the Newcastle-Ottawa Scale for the quality assessment of the included studies. While these are standard practices, there are several areas for potential improvement:

- Choice of Meta-Analytic Model: The fixed-effect model assumes that the effect size is constant across all studies, which might not be the case here given the varied contexts of the studies. A random-effects model could provide a more generalizable estimate.
- Heterogeneity Analysis: It's crucial to assess the heterogeneity among the included studies. If significant heterogeneity is present, exploring its sources (e.g., through subgroup analyses or meta-regression) can provide valuable insights.

**Results**

- The entire result section will need to be formatted to align with the style of other sections of the paper.
- The results are presented with sufficient detail, including the number of studies reviewed and the main findings. To improve this section, authors may provide a more nuanced analysis of the results, including subgroup analyses or meta-regressions if possible.
- Graphical representations like forest plots or funnel plots could be included to visually demonstrate the results and potential biases.

**Conclusion and Recommendation**

- The conclusions drawn align with the objective and results. Recommendations are practical and relevant. However, emphasizing the implications of these findings for future research and public health practice would enhance this section.
